# Hospital Cases

**Published:** 1854-08

**Authors:** John Neill

**Affiliations:** Surgeon to the Pennsylvania Hospital


### MEDICAL EXAMINER.

NEW SERIES.—NO. CXVI.—AUGUST, 1854.

---

### ORIGINAL COMMUNICATIONS.

---

*Hospital Cases.* By JOHN NEILL, M. D., Surgeon to the Pennsylvania Hospital.

From the notes of DR. RHOADS, House Surgeon.

*Rupture of the Urethra without external wound. Perineal Section.*

CASE No. 1.—W. T., a young Englishman, employed upon one of the locomotives of the Reading Railroad, was injured by a collision on the 13th of August, 1852. He was brought to the Hospital on the next day, and, upon examination, was found to be much bruised about the pelvis and perineum. Although he was astride of the brake at the time the accident occurred, no external wound had been made; there was not even an abrasion of the skin.

No fracture of the bones of the pelvis could be detected; there was neither crepitus, displacement nor paralysis, except of the bladder; he had passed no urine since the accident. Upon attempting to pass a catheter I discovered that it was ruptured at its membranous portion, and from the sensation communicated to the catheter, felt confident that the laceration was not a slight slit but an extensive division of this portion of the urethra; blood issued from the catheter, but of course no urine. After careful manipulation with the instrument in search for the extremity of the short part of the urethra, and failing to reach the bladder, I concluded to make a section of the perineum before the bladder should become distended and before infiltration should take place.

*Operation.*—A catheter was introduced into the urethra as far as the seat of rupture, to serve as a guide, and a free deep incision made in the mesial line. All of the tissues were completely infiltrated with blood, and the end of the catheter was found, after slight dissection, completely free from the urethra which had been torn completely across. There was not even a connecting shred left between the two portions of the urethra to serve as a guide from one portion to the other. Not being able to discover the orifice of the prostatic portion, the cellular tissue being so completely infiltrated with blood, I determined to puncture the bladder through the prostate gland. This was accomplished by means of a conical silver catheter, though not without difficulty, on account of the displacement of the parts and the numerous coagula.

When the bladder was punctured the urine flowed freely to the amount of 12 oz., and the patient was much relieved. The catheter was secured in this position, and lint placed between the lips of the wound in order that the extravasated blood might make its escape. ℞. Sol. Morph. Sulph. f.ʒij.

*Aug.* 14. Slept tolerably, well but complains of supra-pubic and abdominal pains. Directed warm fomentations; morphia at night.

*Aug.* 15. Bowels not yet opened. ℞. Rhei et Cal. *aa* gr. x.; light nourishment.

*Aug.* 16. Pain continues. Leeches over the pubis; enema of castor oil. The wound, after being poulticed, was carefully washed and dressed with wet lint. Much clotted blood has escaped, and now the surfaces seem disposed to granulate. The urine escapes regularly through the catheter.

*Aug.* 17. Less abdominal pain, but complains of pain in the penis and desire to urinate, though the urine still flows freely. His bowels are opened regularly.

*Aug.* 18. Has a comfortable skin and pulse, but still has pain and hardness over the bladder.

*Aug.* 20. To-day there was some hæmorrhage from the wound; blood also escaped from the penis. After cleaning the wound the orifice of the urethra was searched for and discovered by the urine oozing from it. A female catheter was introduced into the bladder from the wound, and the conical catheter was withdrawn from the opening which was originally made by its forcible introduction through the prostate. A male catheter was introduced through the penis and crossed the female catheter in the wound; afterwards a slight hæmorrhage.

*Aug.* 21. Hæmorrhage occurred again. Pulse feeble and quick, skin pallid. Directed compression with lint and ice. Stimulus.

*Aug.* 22. Blood passed through male catheter last night freely. Treatment continued.

*Aug.* 23. Skin moist and sallow, pulse feebler; complains of no pains but that of the penis. Great prostration. Died at 10 o'clock.

*Post mortem, Aug.* 24. Upon opening the cavity of the abdomen a large cavity of pus was exposed, which had formed between the peritoneum and the muscular parietes of the abdomen, and which probably had been the occasion of his supra-pubic and abdominal pain. The peritoneum revealed no evidence of inflammation; it contained a small amount of serum tinged with the coloring matter of the blood.

The bladder contained a large amount of coagulated blood.

Upon examination of the wound in the perineum, several fractures of the pelvis were discovered which had not been detected before death. Both rami of the pubes and the ramus of the ischium were fractured and impacted, and large collections of pus, formed in the vicinity, had extended between the symphisis pubis and the bladder, and throughout the pelvis fascia towards the abdomen.

*Remarks.* In the case related above we have a striking illustration of the fact that serious fractures of the pelvis may take place and give no positive evidence of the injury. This patient was carefully examined for fracture, for the severe nature of the accident and the rupture of the urethra both led to the suspicion of that accident, but there was neither mobility, crepitus, nor deformity; and no paralysis, except of the pelvic viscera, which often occurs from other injuries. Although the pelvis was broken in four different places, nevertheless, by the impaction of the fragments, the anterior arch of the cavity was so preserved, that no irregularity could be detected by the finger, through the wound, when firmly pressed against the arch of the pubis. The position and retention of the fragment makes it certain that the rupture of the urethra was not the result of the fracture; it could not have been torn by a rough sharp edge of bone, for there was no displacement. There must have been some other force, and it is a matter of some interest to determine what this force is. Rupture of the urethra and laceration of the bladder are unquestionably often produced by projecting portions of a fractured pelvis. But I suspect that many cases of hæmorrhage from the urethra which are often attributed to fractures, are the result of force producing laceration; and yet, at first sight, it is not very evident how this force is to be directed to produce such a result. When we reflect upon the structures of the perineum, the elasticity of the skin, the thickness of that portion of superficial fascia in front of the weakest portion of the urethra, and the depth of this part of the urethra, we certainly do not see how any blow, however severe, would be followed by laceration and hæmorrhage. Would it not be natural to suppose that such soft, cushion-like parts would recede before the blow, and that these organs retiring towards the abdomen would be secure? Unless the instrument is pointed, and driven directly towards the membranous portion, we do not see why the urethra should be so frequently torn as it passes through the triangular ligament. It is admitted by many authorities that this is the portion most frequently torn, and yet it is certainly not the most exposed, therefore we must look for an explanation of this fact.

I believe it may be found in an investigation of the action of muscles of the perineum. A glance at a drawing of muscles of the perineum will show this portion of the urethra to be the seat of opposing muscular contractions. The centre of the letter X will serve to represent the membranous part of the urethra as it passes through the triangular ligament, and the two converging lower limbs of the letter will represent the line of action of a portion of the sphincter ani, levator ani, and transversalis perinei muscles, whilst the two upper limbs correspond to the fibres of the accelerator urinæ, and represent the line of their action. Now, let us suppose a blow to be received upon the perineum, no matter whether the blow be made with a blunt or pointed instrument, and whether it be directed accurately against this point of the urethra or not, we see that, in addition to the irregular action of the direct force, that there is acting conjointly a force of muscular contraction excited by the blow. The skin of the perineum is very sensitive, and a slight blow will produce muscular contraction by reflex action. The muscles of the perineum just mentioned will draw the prostate upwards and backwards, and the accelerator urinæ will draw the bulb forward, which may in some measure, at least, account for laceration of the urethra at the membranous portion, even when there is no fracture and no external wound.

These remarks do not pertain to this case just stated, but have been suggested by it and other cases of a somewhat similar nature.

There is, however, a practical point to which I would call the attention of those who may be placed in a similar position. After I had introduced the female catheter into the bladder through the prostatic portion of the urethra, and the male catheter through the penis, the instruments crossed each other in the wound, and there was no mode of joining them so as to make a continuous tube. I removed both, and made use of a flexible leaden catheter; after introducing it through the penis I pulled it out of the wound, and then directed the point of it through the shorter portion towards the bladder. The instrument I used was too short, and I failed in carrying out my design.

Subsequently I found in Mr. Skey's Operative Surgery, a work which certainly contains many valuable suggestions, directions for this same procedure; he recommends the ordinary gum catheter, and gives a wood cut (No. 73,) to which I would refer all who may be interested in the surgery of the perineum.

The next two cases are reported for the purpose of proving that the result in serious compound and complicated fractures of the face is more satisfactory than is generally supposed.

It may not be amiss to remark that these injuries are not generally detailed very fully in some of our most valuable text books. The subject is often dismissed with a single paragraph, and in other instances with a few general remarks, and a reference to the report of a cure published in some other work.

*Fractures of the bones of the Face.*

Case No. 2.—Bernhard Pelster, æt. 22, was admitted into the Hospital 8mo. 11, 1852.

While looking up the hatchway of a large sugar house, he was struck upon the right cheek by a mould weighing 15 pounds, which had fallen from the eighth story of the building.

The great force of the blow, beside lacerating the integuments, caused a compound comminuted fracture of the bones of that side of the face. The alveolar and palate processes of the upper maxillary were broken, and the fragments driven downwards and inwards, so as to destroy the lines of curvature of the teeth and roof of the mouth; the nasal process was also broken, and so separated as to permit the nasal cavity to communicate with the wounds of the cheek. The part of the malar bone forming the lower margin of the orbit was crushed, driven inwards, and the eye slightly forced upwards. A portion of the alveolar ridge of the lower jaw, in which the right central and lateral incisors were inserted, was split off and hanging by a small pedicle of the soft tissues. There was a very extensive and irregular lacerated wound of the cheek. The upper lip was completely severed, the rent passing above the ala of the nose, which was torn loose at its lower and outer part. A wound communicating with the fracture beneath existed just below the orbit of the right eye.

The bony fragments were adjusted as far as possible, and those of the lower jaw retained in position by wire passed around the adjacent sound teeth. The ragged flaps of integument were brought together by a few twisted sutures and adhesive strips, and cold water dressing applied.

8*mo*. 12. Slept little; much swelling of the injured parts; has headache; pulse 60; bowels confined. Ordered a purgative enema, an opiate at night, and a liquid nourishing diet; continue cold water dressing.

8*mo*. 14. Says he feels much better; the swelling of the face has somewhat subsided, and suppuration has commenced from the wounds. He slept badly, however, and is this morning restless, talkative and slightly delirious; pulse good; skin moist and relaxed; has incipient mania-a potu. Ordered brandy punch f.℥j., beef essence f.℥ij. and liq. morph. sulph. f.ʒij., each every three hours.

8*mo.* 15. Slept during the night and is better this morning. Diminish the morphia and stimulants gradually; poultice to the face.

After this date he steadily improved, although extensive suppuration occurred among the bony fragments and lacerated soft parts. The sutures first introduced cut out without producing much adhesion, and after a clean granulating surface had formed, two others were applied to close the fissure in the lip and retain the ala of the nose in position. These were followed by extensive union, and greatly diminished the deformity.

8*mo.* 25. The external wounds have healed. The upper row of teeth and roof of the mouth are still irregular. The small fragment of the lower jaw is still quite movable.

9*mo.* 28. He was discharged cured. The irregularity in the position of the teeth of the upper jaw diminished very much as the cure progressed, so as finally to interfere very little with mastication. The piece of the lower jaw which was at first hanging by a small pedicle, became firmly adherent to the bone, and the teeth useful in mastication. A depression remained in the cheek, but the deformity was not striking, and greatly less than might have been anticipated at the time of his admission.

(There was an interesting complication in this case, when he first entered the house. He had severe contusion of the right shoulder and front of the neck, with complete paralysis of the muscles of the shoulder and arm, in consequence of which the head of the humerus was allowed to fall the full length of the capsular ligament, giving rise to the appearance of a dislocation. Under treatment the paralysis almost completely disappeared, and with it the simulated dislocation of the humerus.)

Case No. 3.—J. B., æt. 20, was admitted 3d mo. 4, 1852.

While in a stable, drunk, and fighting with another man, he was knocked down and, as he supposed, trodden upon by the horses. There were several ragged wounds upon his face; one above the left eye, severing the brow from the forehead, and allowing it to fall over the eye; another upon the left side of the nose an inch and a half long, laid bare the nasal bone, which, however, was not fractured; two others upon the right cheek beneath the orbit communicated with a fracture of the right upper maxillary bone. The alveolar processes, which sustained the molar and last bicuspid teeth, were separated from the body of the bone, the palate process was fractured to a corresponding extent, and the fragment carried towards the centre of the cavity of the mouth, so as to bring the teeth into its central line.

It was found impossible to restore the fragment completely to its natural position, as firm pressure gave rise to free bleeding without entirely overcoming the displacement. Cold water applied by lint to the face.

3*mo.* 5. The oozing of blood had ceased; the fragment was still farther replaced and retained in position by wire passed around the sound teeth.

3*mo.* 18. The wounds have nearly healed, excepting those communicating with the seat of fracture, which show a disposition to become fistulous, although they do not give exit to any saliva. They are to be touched occasionally with caustic.

He continued slowly to improve till the 26th, when he was attacked with pneumonia, from which, though early recognized and actively treated, the convalescence was very protracted.

He was discharged cured. The fragment of the jaw remained displaced inwards to the extent of half an inch, notwithstanding every effort by pressure and support to restore it to its natural position. A small depression existed in the right cheek beneath the orbit.

CASE NO. 4.—Bernard Devlin, æt. 28, wagoner, was admitted 5mo. 15, 1853, at $1\frac{1}{2}$ A. M.

About 5 P. M. of the 14th, while intoxicated, he fell beneath his wagon, which contained three tons of coal, and the wheel passed over his head, his right hand and the outer part of the right thigh.

On admission he was very prostrate; the pulse 120, small, feeble and quick; the skin cool; the pupils dilated and insensible to light; and he lay as if in a deep sleep, with slightly sertorous breathing. When roused he gave very imperfect signs of hearing questions, moved his body and limbs, and evinced sensibility to pain. Sinapisms and artificial heat were applied, an enema of oil of turpentine ordered, and his wounds temporarily dressed.

At 9 A. M., contrary to expectation, he was alive and more conscious, pulse slower and less feeble; he was very restless. A careful examination revealed a comminuted fracture of the superior maxillary and malar bones of the right side, and of the frontal at the external angular process. The side of the face was flattened, the cavity of the orbit diminished, and the eye protruded. Two small wounds of the temple did not communicate with the seat of fracture. The inferior maxillary was broken in two places; on the left side between the last bicuspid and first molar tooth, the line of fracture extending downwards and backwards; on the right side through the ascending ramus. The middle fragment was drawn downwards and backwards by the muscle attached to it.

The integument of the dorsum of the right hand was forced off and turned down over the fingers, laying bare the extensor tendons. A wound six inches long, and extending deeply among the muscles, existed upon the outer side of the right thigh.

The fragment of the upper jaw was pushed back into its natural position, and there retained by wire around the teeth. Those of the lower jaw on the left side were also similarly adjusted and retained by wires.

The wounds of the hand and thigh were dressed; cold water applied to the scalp and face. Has retention of urine; catheterization.

7½ P. M. He is less sensible and very restless; pulse 92; skin hot and dry. ℞. Hyd. chl. mit., pulv. jalapæ *aa* gr. x. at once. ℞. Liq. potas. citrat. ℥ss., vin. antimonii gtt. x. every two hours. Barley water diet.

5*mo.* 16. Passed a restless night; pupils still dilated; intelligence better; pulse 80; skin less hot; bowels purged; continue medicine and cold to head.

5*mo.* 17. Was worse towards evening yesterday, but has had a good night; intellect quite clear; pulse and skin nearly natural. Wounds to be dressed with poultices.

*5mo.* 21. Has continued to improve, though he has been occasionally a little delirious, and has had evening exacerbations of fever every day till to-day; pupils still a little dilated; the frontal bone is now seen to be broken within the external angular process, and from the protrusion of the eye and ecchymosis of the orbit, the orbitar plate is also probably involved. The fragment of the upper jaw is in place; those of the lower jaw are in contact upon the left side, but no bandage can be used to keep them in position, and the middle fragment falls downwards. The sloughs are separating from the wounds of the hand and thigh. He has continued the mixture, which keeps his bowels freely open; suspend it.

*5mo.* 31. For the last three days he has complained of his right eye, which has been attacked with conjunctivitis. An abscess has formed at the left fracture of the lower jaw, and has opened into the buccal cavity. Poultice to abscess.

*6mo.* 2. The abscess of the jaw, now pointing externally, was opened to-day.

*6mo.* 7. Since last notes the projection of the eye and pain in the orbit have disappeared, the subsidence of the symptoms coinciding, he says, with the discharge of pus into the back part of the nasal cavity. This morning he has again pain in the orbit, protrusion of the eye ball, strabismus and severe headache. Ordered cut cups to the back of the neck, and some purgative pills, which measures quickly relieved him. The abscess around the fracture of the lower jaw has nearly healed.

After this date Barton's bandage was applied, and although union had not occurred, no force that could be applied was sufficient to overcome the displacement downwards of the anterior fragment. Some small pieces of bone were exfoliated through the abscess. The eye was protruded and again subsided several times, each subsidence being attended with discharge of pus from the orbit into the nose.

He was discharged cured 8th mo. 29, 1853. Some flattening of the right malar prominence remained; the eye was rather prominent, its pupil dilated, and vision with it quite imperfect. The fracture of the upper jaw healed without deformity. Firm union took place in the lower jaw, but he was unable to approximate the teeth perfectly in front; he could, however, masticate food readily with the back teeth.

